# Errata

**Published:** 1869-03-15

**Authors:** 


### *Errata.*

In Dr. Wallihan's article on Super-Oxygenation, commenced in the number for February 15th, page 117, instead of "late investigations," read "later investigations."

Page 119, for "permanently Nature's own," read "preeminently," etc.

Page 140, in the sentence "Sprague, of Boston," etc., insert "out" after "brought," and omit "polished;" and before "*Protoxide of nitrogen*" read "2" instead of "1".

Page 141, for "neutralizing" read "neutralize."

Page 143, insert "as" before "unfit," and read "experimenters" instead of "experimenter."

Page 145, for "attractive, antiseptic," etc., read "alterative, antiseptic," etc.

Page 146, for "varo gas" read "raw gas."

Page 148, for "usual complication" read "renal complication."

Page 150, Case 4, read "feeble pulse and weak digestion" instead of "feeble and weak digestion."

